# Neutrophil recruitment and activation are differentially dependent on MyD88/TRIF and MAVS signaling during RSV infection

**DOI:** 10.1038/s41385-019-0190-0

**Published:** 2019-07-29

**Authors:** Freja C. M. Kirsebom, Fahima Kausar, Rinat Nuriev, Spyridon Makris, Cecilia Johansson

**Affiliations:** 1 National Heart and Lung Institute, Imperial College London, St Mary’s Hospital, Norfolk Place, London, W2 1PG UK; 2 Present Address: MRC/UCL Lab for Molecular Cell Biology, London, UK

## Abstract

Respiratory syncytial virus (RSV) is a leading cause of severe lower respiratory tract infections, especially in infants. Lung neutrophilia is a hallmark of RSV disease but the mechanism by which neutrophils are recruited and activated is unclear. Here, we investigate the innate immune signaling pathways underlying neutrophil recruitment and activation in RSV-infected mice. We show that MyD88/TRIF signaling is essential for lung neutrophil recruitment while MAVS signaling, leading to type I IFN production, is necessary for neutrophil activation. Consistent with that notion, administration of type I IFNs to the lungs of RSV-infected *Mavs*^*−/−*^ mice partially activates lung neutrophils recruited via the MyD88/TRIF pathway. Conversely, lack of neutrophil recruitment to the lungs of RSV-infected *Myd88/Trif*^*−/−*^ mice can be corrected by administration of chemoattractants and those neutrophils become fully activated. Interestingly, *Myd88/Trif*^*−/−*^ mice did not have increased lung viral loads during RSV infection, suggesting that neutrophils are dispensable for viral control. Thus, two distinct pathogen sensing pathways collaborate for neutrophil recruitment and full activation during RSV infection.

## Introduction

Neutrophils are classically considered as among the first immune cells to respond to both infection and injury. Historically, neutrophils were considered as non-specific effector cells but it is becoming increasingly clear that they can respond differentially to harmful stimuli.^1^ Pattern recognition receptor (PRR) signaling induces innate immune responses including the generation of chemotactic gradients along which neutrophils are recruited into affected tissues.^2,3^ Following recruitment, additional signals are required to trigger neutrophil activation and degranulation. During activation, neutrophils can secrete hundreds of pre-stored effector proteins and proteolytic enzymes including matrix metalloproteinases (MMPs), myeloperoxidase (MPO), and neutrophil elastase (NE).^4^ Neutrophils can also drive inflammation by generating reactive oxygen species (ROS), as well as by undergoing a unique form of cell death whereby they secrete nuclear DNA in the form of neutrophil extracellular traps (NETs).^4–6^ Whilst neutrophils are important as immediate antimicrobial responders, their recruitment and activation must be tightly regulated to minimalize pathology caused by bystander effects on host tissues.^7,8^ This is particularly relevant in the lung where the disruption of delicate alveolar structures can rapidly have life-threatening consequences if gas exchange is compromised. The molecular mechanisms regulating neutrophil recruitment and activation in response to bacterial and fungal respiratory infections have been well characterized.^9^ However, considerably less is known about the regulation of neutrophil action during respiratory viral infections.^10–12^

Respiratory syncytial virus (RSV) is the greatest cause of infant hospitalizations in the developed world.^13,14^ More than 90% of children encounter RSV within their first 24 months and a minority develops severe disease; 1–3% will require hospitalization.^15^ In addition to acute disease, RSV-induced bronchiolitis and viral pneumonia in infancy are associated with debilitating, long-term respiratory disorders, such as recurrent wheeze^16^ and asthma.^17^ Although it is not well understood why some infants are asymptomatic while other infants develop severe disease, host immune factors have been implicated in disease severity.^18,19^ During RSV infection, neutrophils are recruited to the lungs of both mouse and man.^20–22^ Clinical studies suggest that neutrophils contribute to immune pathology during disease^20,22,23^ and they are the most abundant cell type in the bronchoalveolar lavage (BAL) of infants with RSV-induced bronchiolitis.^20,22^ Neutrophil activation during RSV infection has been more difficult to assess clinically than neutrophil recruitment. However, elevated NE concentrations have been observed in both the serum and nasal lavage of children with confirmed RSV infection.^23^ Together, these studies suggest that a hyperactive or dysregulated neutrophil response to RSV may underlie disease severity and indicate the importance of studying the signaling pathways that regulate neutrophil recruitment and activation in the infected lung.

Innate immune receptors on epithelial cells and alveolar macrophages (AMs) initiate the immune response to RSV within the respiratory tract.^24,25^ The plasma membrane expressed Toll-like receptors (TLRs), TLR2/TLR6 and TLR4, and the endosomally-expressed TLR3 and TLR7, have all been implicated in RSV detection.^25,26^ RSV is also detected in the cytosol by retinoic acid-inducible gene I (RIG-I)-like receptors (RLRs): RIG-I and melanoma differentiation–associated protein 5 (MDA5).^27–29^ RLR signaling occurs through the adaptor protein mitochondrial antiviral signaling protein (MAVS)^30,31^ while TLRs signal via the adaptor proteins MyD88 and/or TRIF.^32^ Signaling via MAVS, MyD88 and TRIF induces the expression of type I and type III interferons (IFNs), as well as other pro-inflammatory cytokines and chemokines.^33^ Type I IFNs bind to the interferon-α/β receptor (IFNAR) expressed on all nucleated cells and induce the expression of hundreds of IFN-stimulated genes (ISGs).^34^ ISG protein products have direct antiviral properties and can also promote the recruitment and activation of other components of the immune response, including antiviral monocytes.^21,27^

In this study, we investigated the necessity of MAVS and MyD88/TRIF dependent signaling pathways to lung neutrophil recruitment and activation during RSV infection in vivo. *Mavs*^*−/−*^ mice, unable to signal via MDA-5 or RIG-I, and *Myd88/Trif*^*−/−*^ mice, unable to signal via TLRs (and the IL-1 receptor (IL-1R) and IL-18 receptor (IL-18R)) were infected with RSV. We found that neutrophil recruitment to the lung was dependent on MyD88/TRIF signaling but mostly independent of MAVS signaling. The neutrophil chemoattractant CXCL1 was shown to be mainly produced by non-epithelial, non-endothelial (CD45^−^, Epcam^−^, CD31^−^) lung cells. However, neutrophil activation in the lung was dependent on the type I IFN-driven, pro-inflammatory lung environment induced by MAVS signaling. Restoring the pro-inflammatory environment in the lungs of *Mavs*^*−/−*^ mice using recombinant IFN-α (rIFN-α) was sufficient to partially activate neutrophils during RSV infection. Conversely, neutrophils recruited into the lungs of *Myd88/Trif*^*−/−*^ mice using recombinant CXCL1 (rCXCL1) became fully activated during RSV infection. This study sheds light on the molecular mechanisms that drive neutrophil recruitment and activation in the lungs during RSV infection and demonstrates how two distinct PRR signaling pathways collaborate to drive neutrophil responses to virus infection.

## Results

### RSV infection is characterized by early neutrophil recruitment and activation in the lung

**Fig. 1:**
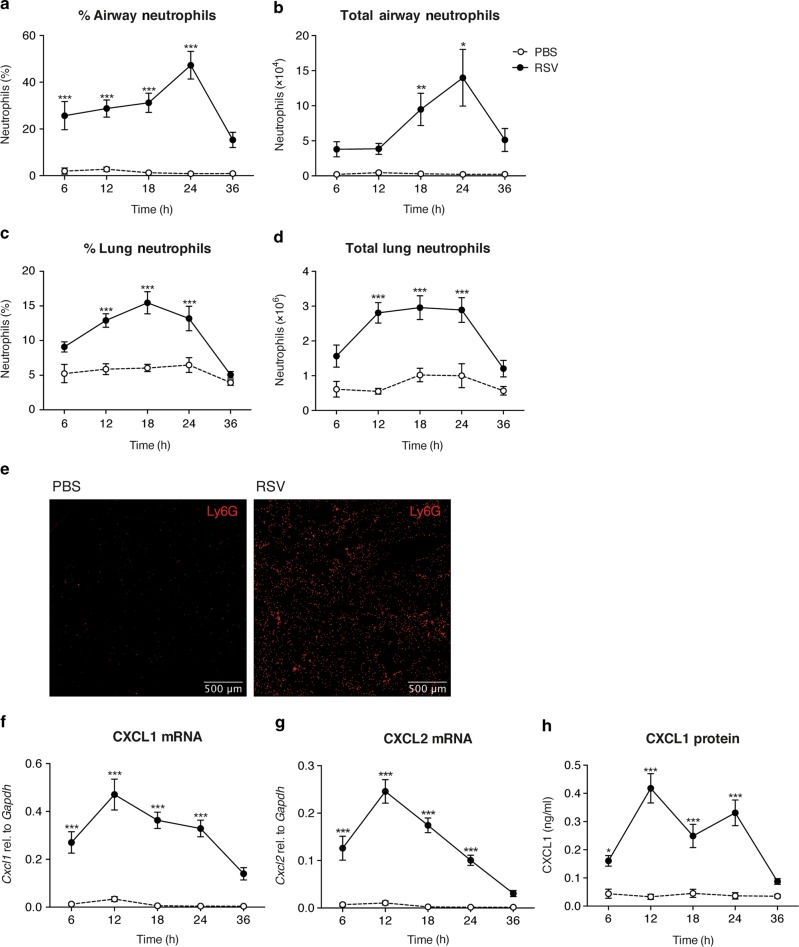
Neutrophil recruitment to the lung during RSV infection in vivo. Wt mice were intranasally mock (PBS) or RSV infected. **a**–**d** Frequencies and total numbers of CD45^+^, CD3^−^, CD19^−^, MHC-II^−^, Ly6G^+^ neutrophils were quantified in the airways and lung using flow cytometry at the indicated time-points (Supplementary Fig. 1a for gating strategy). **e** Images show representative immunostaining of Ly6G^+^ neutrophils (red) in lung cryosections at 24 h p.i. from three mice per group. **f**, **g** *Cxcl1* and *Cxcl2* was quantified relative to *Gapdh* by RT-qPCR from RNA isolated from lung tissue. **h** CXCL1 was detected in the BAL fluid by ELISA. Data are presented as the mean ± SEM of 3–5 (PBS), or 8–13 (RSV) individual mice, pooled from at least two independent experiments. Statistical significance of differences between mock and RSV infection was determined by one-way ANOVA with Tukey’s post hoc test. *P ≤ 0.05, **P ≤ 0.01, ***P ≤ 0.001

Neutrophils are recruited to the lungs during RSV infection in both mice and man.^20,23,27^ To establish the exact timing of neutrophil recruitment to the airways and lungs in the C57BL/6 mouse model, mice were intranasally (i.n.) infected with RSV. The frequencies and total numbers of neutrophils were quantified over time in the airways, by sampling the BAL, and in the lungs using flow cytometry (Fig. 1a–d). Neutrophils were defined as CD45^+^, CD3^−^, CD19^−^, MHC II^−^, Ly6G^+^ cells (gating strategy shown in Supplementary Fig. 1). Neutrophil recruitment to the airways and to the lung peaked at 18–24 h post infection (p.i.) and was not induced by mock (PBS) infection (Fig. 1a–d). Fluorescence microscopy of lung sections demonstrated that Ly6G^+^ cells were evenly distributed throughout the lungs during RSV infection at 24 h p.i. (Fig. 1e). CD64^+^ inflammatory monocyte recruitment to the lung also occurred early following RSV infection (Supplementary Fig. 2a, b), with their recruitment peaking after lung neutrophilia has resolved at 48 h p.i.,^27^ but, in contrast, RSV infection did not have much of an impact on the number of AMs in the airways at any of the early timepoints (Supplementary Fig. 2c). Expression of the neutrophil chemoattractants *Cxcl1* and *Cxcl2* peaked prior to neutrophil influx at 12 h p.i. and this was confirmed at the protein level for CXCL1 (Fig. 1f–h).

**Fig. 2:**
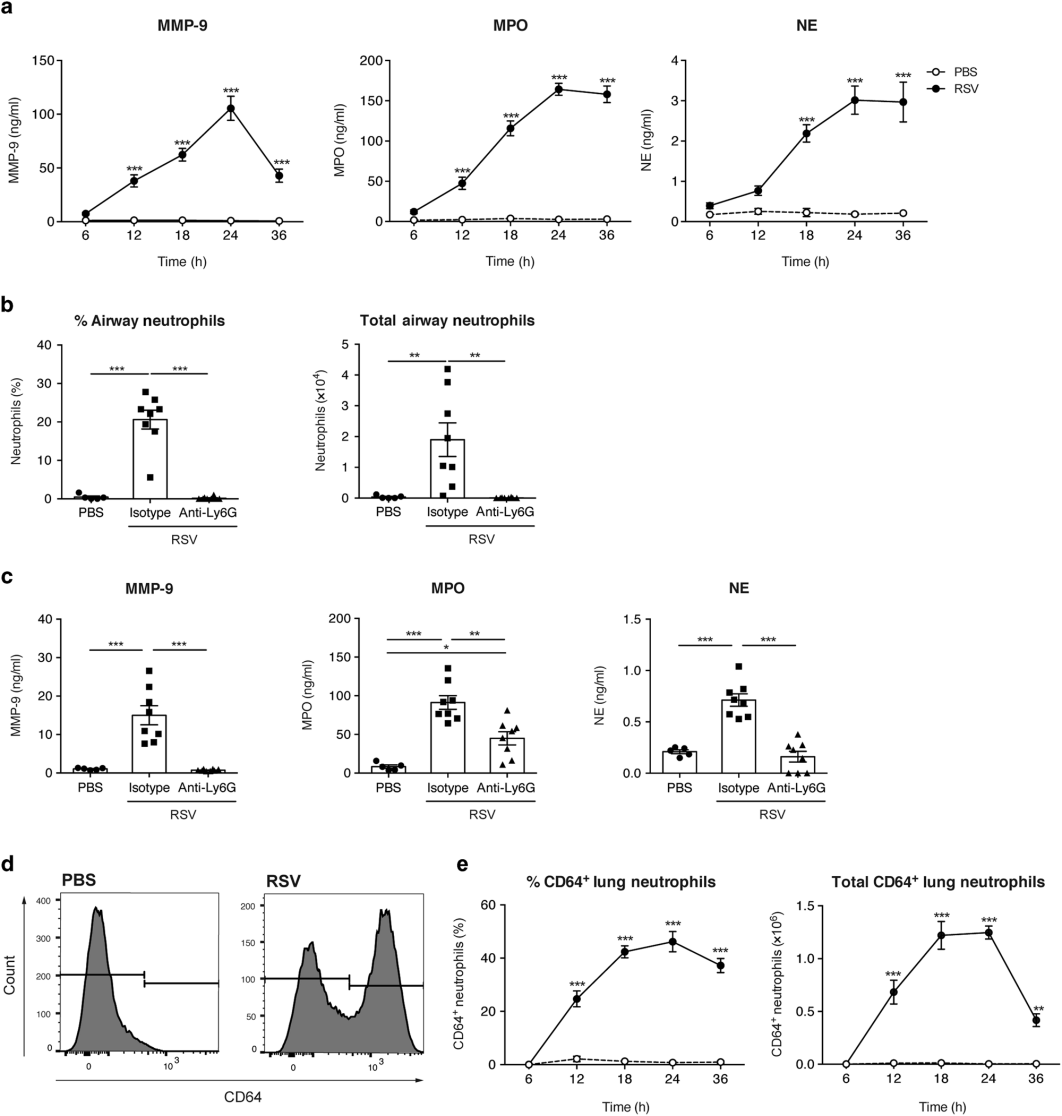
Neutrophil activation in the lung during RSV infection in vivo. Wt mice were intranasally mock (PBS) or RSV infected. **a** MMP-9, MPO and NE were detected in the BAL fluid by ELISA. **b**, **c** Mice were given 200 μg i.n. and 500 μg i.p. α-Ly6G or isotype control antibody 1 day before infection. After 18 h p.i., **b** frequencies and total number of neutrophils in the airways were quantified (the frequencies of neutrophils were determined by differential counting of >300 cells on H&E stained cytospin slides) or **c** MMP-9, MPO and NE were detected in the BAL fluid by ELISA. **d** Representative histograms of CD64 expression on CD45^+^, CD3^−^, CD19^−^, MHC-II^−^, Ly6G^+^ neutrophils in the lung 18 h p.i. (Supplementary Fig. 1a for gating strategy) **e** Frequencies and total numbers of CD64^+^ lung neutrophils (CD45^+^, CD3^−^, CD19^−^, MHC-II^−^, Ly6G^+^), quantified using flow cytometry. Data from the time course are presented as the mean ± SEM of 3–5 (PBS) or 8–13 (RSV) individual mice pooled from two independent experiments. Data from the depletion experiments are presented as the mean ± SEM from 4–5 (PBS) or 7–8 (RSV) individual mice, pooled from two independent experiments. Statistical significance of differences were determined by one-way ANOVA with Tukey’s post hoc test. *P ≤ 0.05, **P ≤ 0.01, ***P ≤ 0.001

Next, we assessed the activation status of neutrophils recruited to the airways and lung during RSV infection (Fig. 2). Neutrophil activation status was initially determined by the detection of the proteolytic enzymes MMP-9, MPO and NE, all of which are pre-stored in cytoplasmic granules and released during neutrophil degranulation.^35^ Whilst neutrophil recruitment peaked at 18–24 h p.i., the secretion of MMP-9, MPO and NE into the airways, as detected in the BAL fluid, peaked at 24 h p.i. (Fig. 2a). Neutrophils were confirmed to be the major cellular source of MMP-9 and NE in BAL as these mediators were not detected after anti-Ly6G depletion (Fig. 2b, c). In contrast, neutrophil depletion with anti-Ly6G reduced but did not entirely abrogate MPO levels in BAL suggesting that there could be additional cellular sources of MPO in the airways after RSV infection (Fig. 2c).

To further assess neutrophil activation status during RSV infection, we quantified the cell surface expression of a variety of markers associated with neutrophil activation in other inflammatory contexts on lung, airway and peripheral blood neutrophils (Supplementary Fig. 3). Upregulation of CD64, CD69 and CD11b on the cell surface of neutrophils has previously been associated with neutrophil activation,^36–38^ as has the downregulation of CD62L and CD182.^39,40^ During RSV infection, we found that CD69 expression remained low on neutrophils in all tissues, regardless of infection status. CD11b was upregulated upon migration into the airways and lungs, but this was also seen in the absence of an active RSV infection. Likewise, CD62L and CD182 were downregulated on airway and lung neutrophils relative to blood neutrophils, but not specifically in response to RSV infection (Supplementary Fig. 3). Of the activation markers tested, CD64 was the most specific marker of neutrophil activation in the lungs (Fig. 2d, e and Supplementary Fig. 3). CD64 expression levels were low on blood neutrophils and high on lung and airway neutrophil during RSV infection (Supplementary Fig. 3), peaking at 18–24 h p.i. (Fig. 2e). We confirmed that mock infection did not induce the secretion of MMP-9, MPO or NE into the airways or the upregulation of cell surface activation marker CD64 at any time point (Fig. 2). We also confirmed that live virus, and not any mediator in the virus preparation, was necessary for neutrophil recruitment and activation as ultra-violet inactivated RSV (UV RSV) did not recruit neutrophils, induce MMP-9, MPO or NE secretion, or drive CD64 cell surface upregulation on lung neutrophils (Supplementary Fig. 4). Also, HEp-2 cell supernatant did not induce neutrophil recruitment (data not shown). Together, these data confirm that neutrophils are a major cell type recruited to the lungs early during RSV infection in the murine model and demonstrate that RSV infection induces neutrophil activation in the lung.

### Neutrophil recruitment is dependent on signaling via MyD88/TRIF

**Fig. 3:**
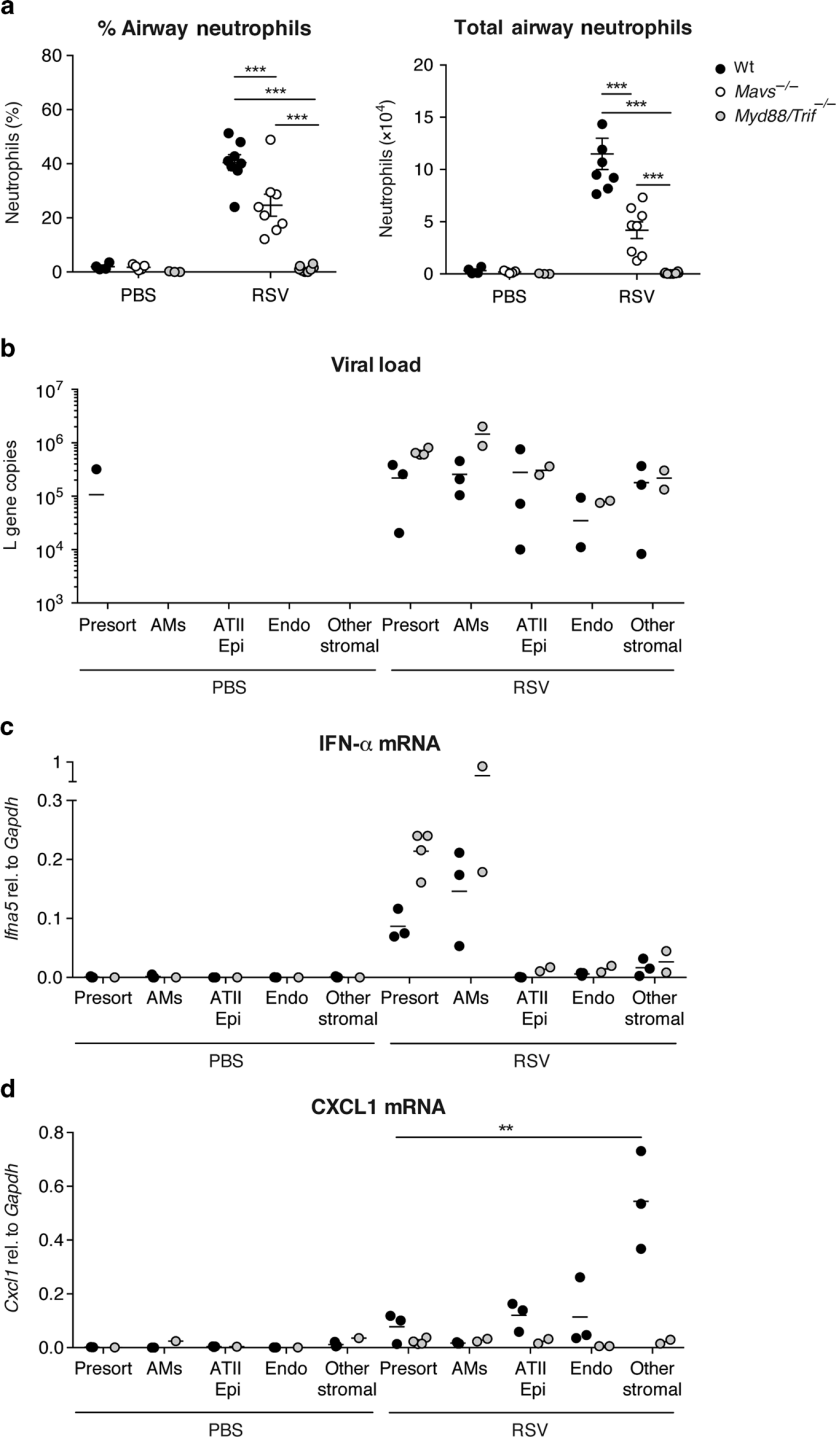
Neutrophil recruitment to the lung and CXCL1 gene induction in stromal cells are dependent on MyD88/TRIF signaling during RSV infection. Wt, *Mavs*^*−/−*^ and *Myd88/Trif*^*−/−*^ mice were intranasally mock (PBS) or RSV infected. **a**, **b** Percentage and total number of neutrophils in the airways 18 h p.i. The frequency of neutrophils was determined by differential cell counting of >300 cells on H&E stained cytospin slides. Data are presented as the mean ± SEM from 3–5 (PBS) or 8 (RSV) individual mice (represented as individual dots) pooled from two independent experiments. **b**–**d** Wt and *Myd88/Trif*^*−/−*^ mice were mock (PBS) or RSV infected for 12 h. RSV L gene copies, *Ifna5* and *Cxcl1* were quantified relative to *Gapdh* by RT-qPCR from RNA isolated from fluorescence activated cell sorted AMs (CD45^+^, CD11c^+^), ATII epithelial cells (CD45^−^, EpCAM^+^, CD31^−^), endothelial cells (CD45^-^, EpCAM^-^, CD31^+^) and other stromal cells (CD45^−^, EpCAM^−^, CD31^−^; Supplementary Fig. 1b for gating strategy) Data are presented as mean ± SEM of 1–3 sorts per group (represented as individual dots), with 3–6 mice pooled per sort. In **a** statistical significance of differences was determined by two-way ANOVA with Tukey’s post hoc test. ***P ≤ 0.001 and in **d** statistical significance of differences between wt sorted cell populations were determined by two-way ANOVA with Bonferroni’s post hoc test. **P ≤ 0.01

RSV is recognized by both RLRs and TLRs, which signal via the cytoplasmic adaptor proteins MAVS and MyD88/TRIF, respectively.^25,26^ To investigate the lack of redundancy of each of these two distinct signaling pathways in neutrophil recruitment, *Mavs*^*−/−*^ and *Myd88/Trif*^*−/−*^ mice were infected with RSV and airway neutrophils were quantified at 18 h p.i. (Fig. 3a). After RSV infection, neutrophils composed 40% of the airway cells in wild-type (wt) mice and 25% of the airway cells in *Mavs*^*−/−*^ mice. No neutrophils were recruited during RSV infection in *Myd88/Trif*^*−/−*^ mice (Fig. 3a). These data indicate that MyD88/TRIF signaling is essential for neutrophil recruitment to the lungs during RSV infection as has previously been implied in *Myd88*^*−/−*^ mice.^41^ As reported,^27–29^ *Mavs*^*−/−*^ mice had a higher viral load than wt mice at 18 h p.i. and at day 4 p.i. (the peak of viral load in the mouse model^21^). In contrast, *Myd88/Trif*^*−/−*^ mice had a similar viral load to wt mice at both timepoints measured (Supplementary Fig. 5).

To further investigate the mechanism driving neutrophil recruitment to the lung during RSV infection, we assessed the source of one of the neutrophil chemoattractants, CXCL1. Wt and *Myd88/Trif*^*−/−*^ mice were mock (PBS) or RSV infected and resident AMs, endothelial cells, ATII epithelial cells and CD45^−^, EpCAM^−^ CD31^−^ cells (other stromal cells) were sorted at 12 h p.i. (Supp. Fig. 1b for FACS gating strategy) and compared to a presorted sample (total lung). RSV L gene transcripts were detected in all cell populations in RSV-infected mice (Fig. 3b). *Ifna5* expression was detected in the total lung sample (presort) and in sorted AMs (Fig. 3c) from both wt and *Myd88/Trif*^*−/−*^ mice, confirming previous findings.^24,27^ Interestingly, *Cxcl1* mRNA was significantly enriched in the CD45^-^, EpCAM^-^, CD31^-^ cell population (other stromal cells) from wt mice, suggesting that non-epithelial, non-endothelial cells are the major source of CXCL1 during RSV infection (Fig. 3d). *Cxcl1* mRNA was not detected in any lung cell populations from RSV-infected *Myd88/Trif*^*−/−*^ mice (Fig. 3d). These data suggest that MyD88/TRIF signaling is crucial for recruitment of neutrophils into the lungs during RSV infection and that signaling via MyD88/TRIF is necessary for inducing *Cxcl1* and, possibly, other neutrophil chemoattractants in the lung stromal cell compartment.

### Neutrophil activation is dependent on signaling via MAVS

**Fig. 4:**
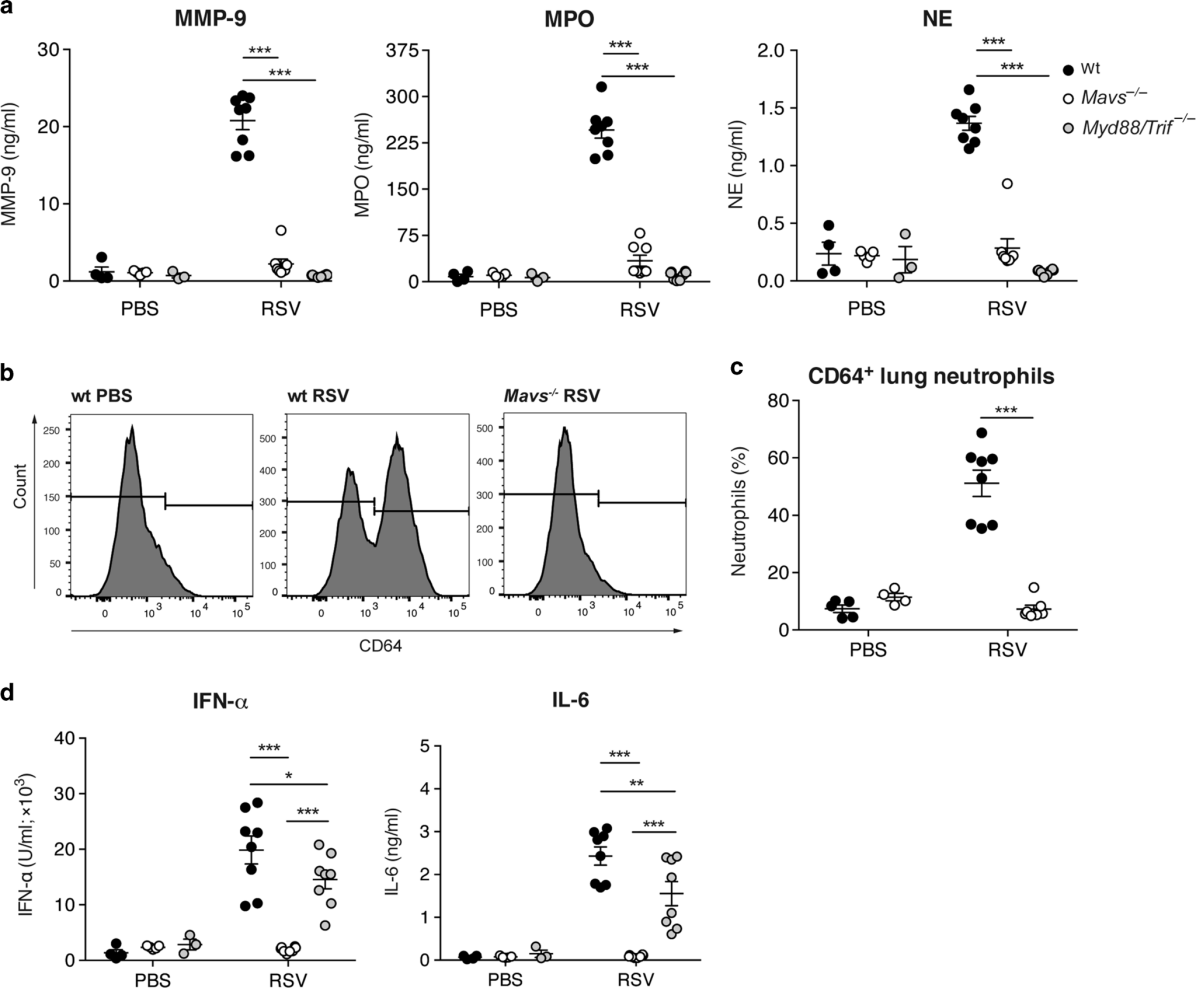
Neutrophil activation is dependent on MAVS signaling during RSV infection. Wt, *Mavs*^*−/−*^ and *Myd88/Trif*^*−/−*^ mice were intranasally mock (PBS) or RSV infected for 18 h. **a** MMP-9, MPO and NE were detected in the BAL fluid by ELISA. **b** Representative histograms of CD64 expression on CD45^+^, Ly6G^+^ neutrophils in the lung. **c** Frequencies of CD64^+^ expression on CD45^+^, Ly6G^+^ lung neutrophils, quantified using flow cytometry. **d** IFN-α and IL-6 were detected in the BAL fluid by ELISA. Data are presented as the mean ± SEM from 3–5 (PBS) or 8 (RSV) individual mice (represented as individual dots) pooled from two independent experiments. Statistical significance of differences was determined by two-way ANOVA with Tukey’s post hoc test. *P ≤ 0.05, **P ≤ 0.01, ***P ≤ 0.001

We next investigated how the MAVS and MyD88/TRIF dependent PRR signaling pathways regulate neutrophil activation in the lung during RSV infection (Fig. 4a–c). MMP-9, MPO and NE were measured in the airways of wt, *Mavs*^*−/−*^ and *Myd88/Trif*^*−/−*^ mice at 18 h p.i. (Fig. 4a). Consistent with their inability to recruit neutrophils to the lung, *Myd88/Trif*^*−/−*^ mice did not display increased levels of MMP-9, MPO or NE in the airways after RSV infection (Fig. 4a). Notably *Mavs*^*−/−*^ mice, which did recruit neutrophils during RSV infection, also did not show elevated MMP-9, MPO or NE in BAL during RSV infection (Fig. 4a). Furthermore, lung neutrophils from RSV-infected *Mavs*^*−/−*^ mice lacked cell surface CD64 expression (Fig. 4b, c). In contrast, the very few lung neutrophils found in RSV infected *Myd88/Trif*^*−/−*^ mice (that have functional MAVS signaling) did upregulate CD64 normally (Supplementary Fig. 6). These data suggest that MAVS signaling is required for neutrophil activation in the lung during RSV infection, at least as measured by CD64 upregulation and the secretion of some granule contents.

Signaling via MAVS is essential for the production of type I IFNs and lung inflammation during RSV infection.^27^ We confirmed that IFN-α and IL-6 were not present in the airways of *Mavs*^*−/−*^ mice at 18 h p.i. (Fig. 4d). However, *Myd88/Trif*^*−/−*^ mice had comparable levels of IFN-α and IL-6 in the BAL to wt mice (Fig. 4d). Therefore, although signaling via MyD88/TRIF is required for neutrophil recruitment during RSV infection, it is not required for the overall establishment of a cytokine-driven, pro-inflammatory lung environment as assessed by IFN-α and IL-6 levels. These observations lead to the hypothesis that, during RSV infection, pro-inflammatory factors induced downstream of MAVS signaling activate lung neutrophils after they are recruited into the lungs as a downstream consequence of MyD88/TRIF signaling.

### Recombinant IFN-α restores the pro-inflammatory lung environment in the absence of MAVS signaling and is sufficient to activate lung neutrophils during RSV infection

**Fig. 5:**
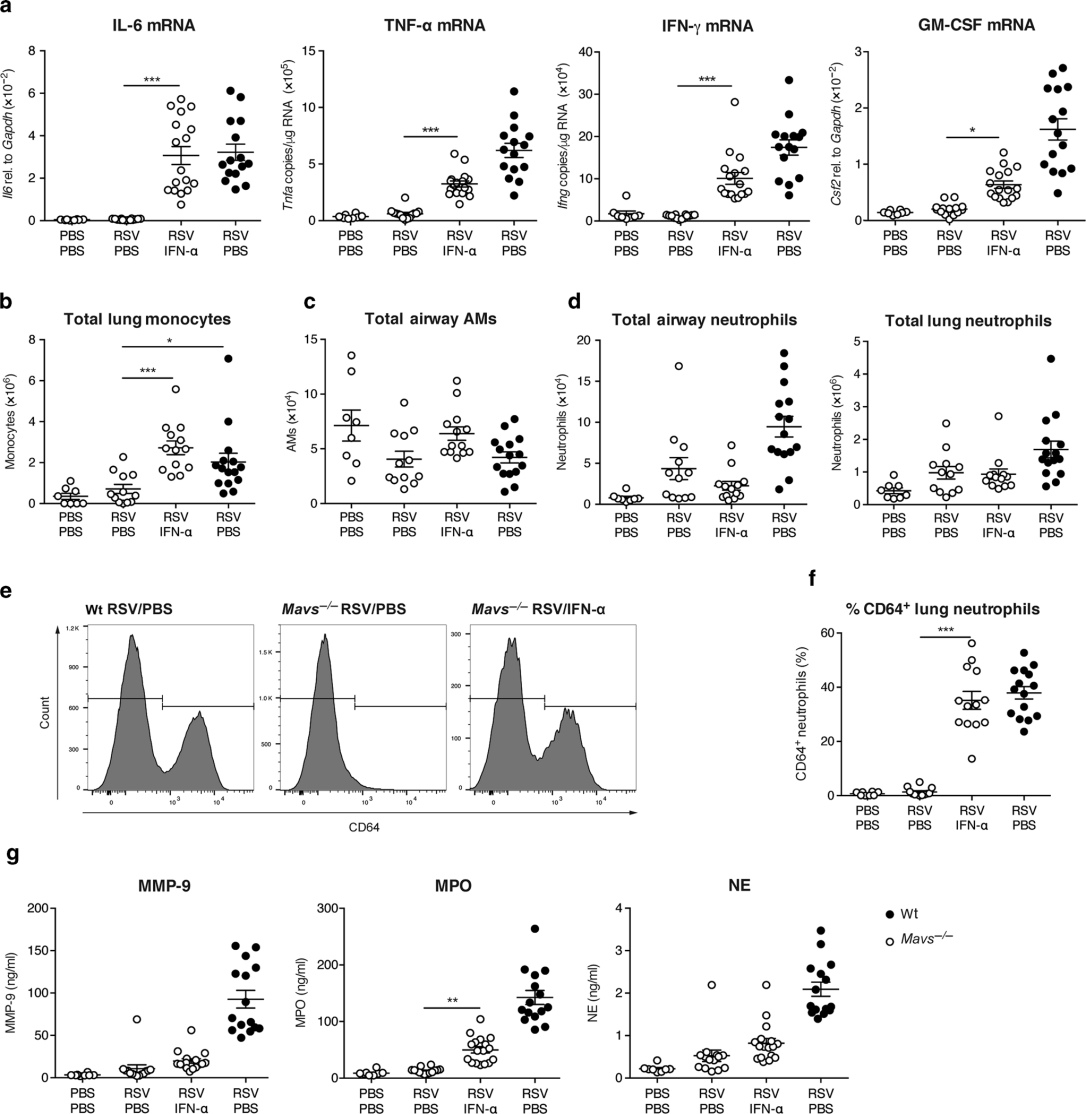
rIFN-α restores neutrophil activation in the lung of MAVS deficient mice during RSV infection. Wt and *Mavs*^*−/−*^ mice were mock (PBS) or RSV infected for 24 h. PBS or 1 μg rIFN-α was administered i.n. 6 h p.i. **a** mRNA was quantified by RT-qPCR from RNA isolated from the lung tissue. Expression levels of *Il6* and *Csf2* were quantified relative to *Gapdh*. Absolute levels of *IFNg* and *Tnfa* were quantified using a plasmid standard. **b** Total number of lung monocytes as quantified by flow cytometry (Supplementary Fig. 1a for gating strategy). **c** Total number of airway AMs as quantified by flow cytometry. **d** Total number of airway and lung CD45^+^, CD3^−^, CD19^−^, MHC-II^−^, Ly6G^+^ neutrophils, as quantified by flow cytometry. **e** Representative histograms of CD64 expression on CD45^+^, CD3^−^, CD19^−^, MHC II^−^, Ly6G^+^ neutrophils in the lung. **f** Frequency of CD64^+^ lung neutrophils, as quantified by flow cytometry. **g** MMP-9, MPO and NE were detected in the BAL fluid by ELISA. Data are presented as mean ± SEM of 8–17 mice (represented as individual dots) from each group, pooled from 3–4 independent experiments. Statistical significance of differences was determined by one-way ANOVA with Tukey’s post hoc test. Only the statistical significances between *Mavs*^*−/−*^ RSV/PBS and RSV/IFN-α are shown. *P ≤ 0.05, **P ≤ 0.01, ***P ≤ 0.001

We tested this hypothesis firstly by restoring the pro-inflammatory environment in the lungs of *Mavs*^*−/−*^ mice during RSV infection by intranasal administration of rIFN-α, which induces pro-inflammatory cytokines and monocyte recruitment in the lungs of wt mice.^21^ We confirmed that this also works in *Mavs*^*−/−*^ mice (Fig. 5a, b) and in mock infected wt mice (Supplementary Fig. 7a) without impacting neutrophil or AM numbers (Fig. 5c, d and Supplementary Fig. 7b). Consistent with induction of pro-inflammatory mediators, rIFN-α but not BSA (a protein control for rIFN-α) was sufficient to cause upregulation of CD64 on lung neutrophils present at baseline in wt mice (Supplementary Fig. 6c). Likewise, rIFN-α was sufficient to drive upregulation of CD64 in RSV-infected *Mavs*^*−/−*^ mice to levels comparable to those in RSV-infected wt mice (Fig. 5e, f). This was accompanied by higher levels of MPO in the BAL (Fig. 5g) and a trend for increased levels of airway MMP-9 and NE, which did not reach statistical significance (Fig. 5g). Thus, rIFN-α is sufficient to drive an inflammatory environment in the lung that results, at least partially, in neutrophil activation in RSV-infected *Mavs*^*−/−*^ mice.

**Fig. 6:**
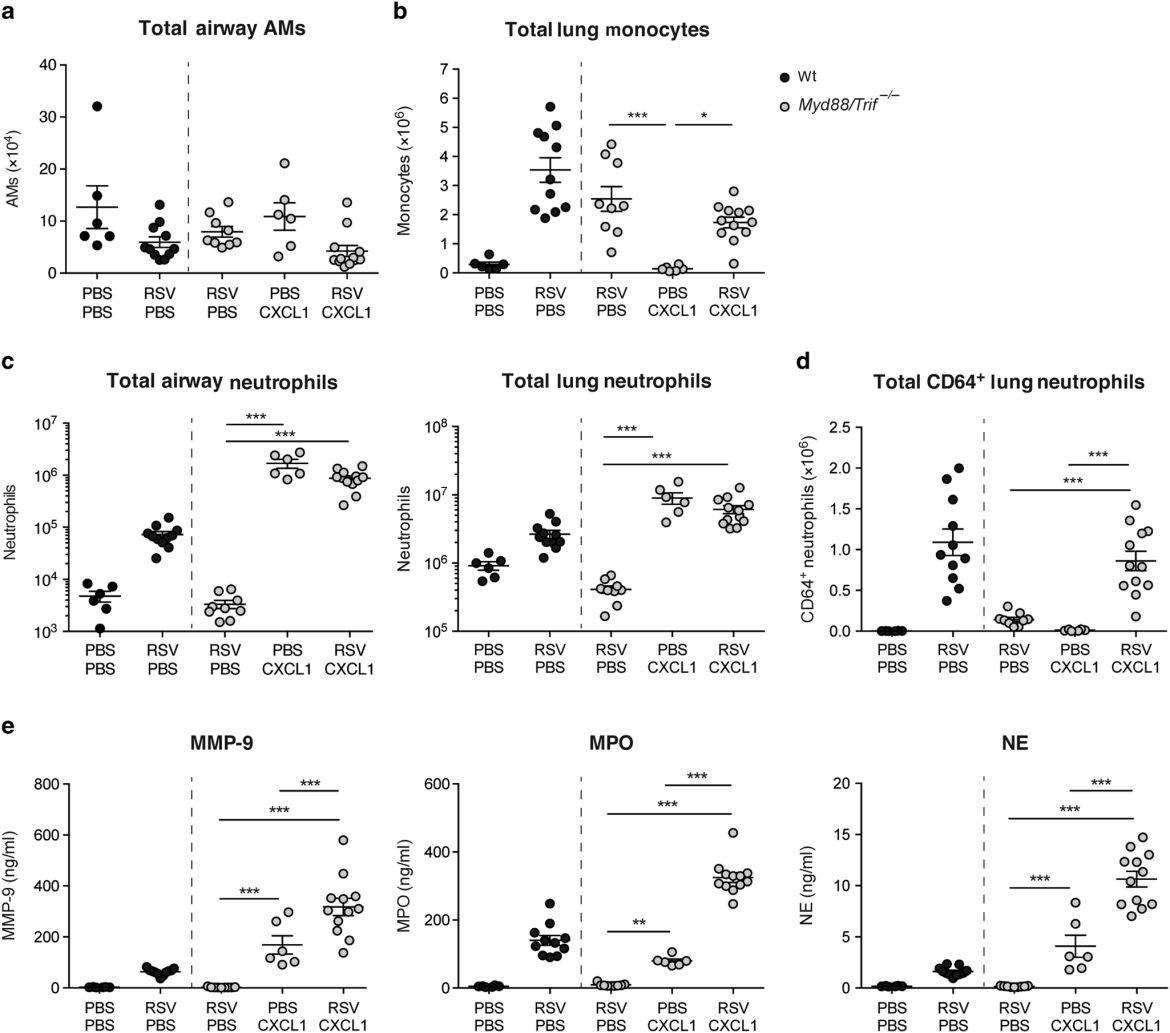
Neutrophil activation is not dependent on MyD88/TRIF signaling in the lung during RSV infection. Wt and *Myd88/Trif*^*−/−*^ mice were mock (PBS) or RSV infected for 18 h. PBS or 10 μg rCXCL1 was administered i.n. 6 h p.i. **a** Total number of airway AMs as quantified by flow cytometry. **b** Total number of lung monocytes as quantified by flow cytometry. **c** Total numbers of airway and lung CD45^+^, CD3^−^, CD19^−^, MHC-II^−^, Ly6G^+^ neutrophils as quantified by flow cytometry. **d** Total number of CD64^+^ lung neutrophils, as quantified by flow cytometry. **e** MMP-9, MPO and NE were detected in the BAL fluid by ELISA. Data are presented as mean ± SEM of 6–12 mice from each group (represented as individual dots), pooled from 2–3 independent experiments. Statistical significance of differences was determined by one-way ANOVA with Tukey’s post hoc test. Only the statistical significances between groups of *MyD88/Trif*^*−/−*^ mice are shown. *P ≤ 0.05, **P ≤ 0.01, ***P ≤ 0.001

Secondly, we used *Myd88/Trif*^*−/−*^ mice to test our hypothesis that factors induced downstream of MAVS signaling activate lung neutrophils during RSV infection. *Myd88/Trif*^*−/−*^ mice produce type I IFNs in response to RSV as they have intact MAVS signaling and therefore have a highly pro-inflammatory lung environment early during RSV infection (Fig. 4). We postulated that *Myd88/Trif*^*−/−*^ mice do produce the neutrophil activating factors in the lung in response to RSV, despite neutrophil recruitment being abrogated. Indeed, as mentioned, the few neutrophils that can be found in RSV infected *Myd88/Trif*^*−/−*^ mice upregulated the activation marker CD64 (Supplementary Fig. 6). However, to further test this hypothesis, *Myd88/Trif*^*−/−*^ mice were treated with rCXCL1 i.n. 6 h p.i. to recruit neutrophils into the lung after RSV infection (Fig. 6). Neutrophil responses were evaluated at 18 h p.i. Treatment of *Myd88/Trif*^*−/−*^ mice with rCXCL1 during RSV infection did not influence the number of airway AMs or lung monocytes as compared to mock treatment (Fig. 6a, b).

As previously observed, *Myd88/Trif*^*−/−*^ mice did not recruit neutrophils to the airways or lungs 18 h p.i. (Fig. 6c) but treatment with 10 μg rCXCL1 was sufficient to recruit neutrophils (Fig. 6c). There was no difference in neutrophil recruitment between mock infected or RSV infected *Myd88/Trif*^*−/−*^ mice treated with rCXCL1 (Fig. 6c). Lung neutrophils recruited into RSV-infected *Myd88/Trif*^*−/−*^ mice displayed an activated phenotype as measured by the upregulation of cell surface CD64, which was comparable to that observed on neutrophils during RSV infection of wt mice (Fig. 6d). CXCL1 treatment in the absence of RSV did not cause upregulation of CD64 on lung neutrophils in *Myd88/Trif*^*−/−*^ mice (Fig. 6d), confirming that the MAVS-dependent inflammatory environment is required for full neutrophil activation. On its own, rCXCL1 did induce the accumulation of some MMP-9, MPO and NE in the BAL of *Myd88/Trif*^*−/−*^ mice (Fig. 6e), but this was significantly increased during RSV infection. Together, these data demonstrate that the factors which activate lung neutrophils during RSV infection are present in the lungs of *Myd88/Trif*^*−/−*^ mice despite the fact that these mice cannot recruit neutrophils to the lung during the infection.

## Discussion

**Fig. 7:**
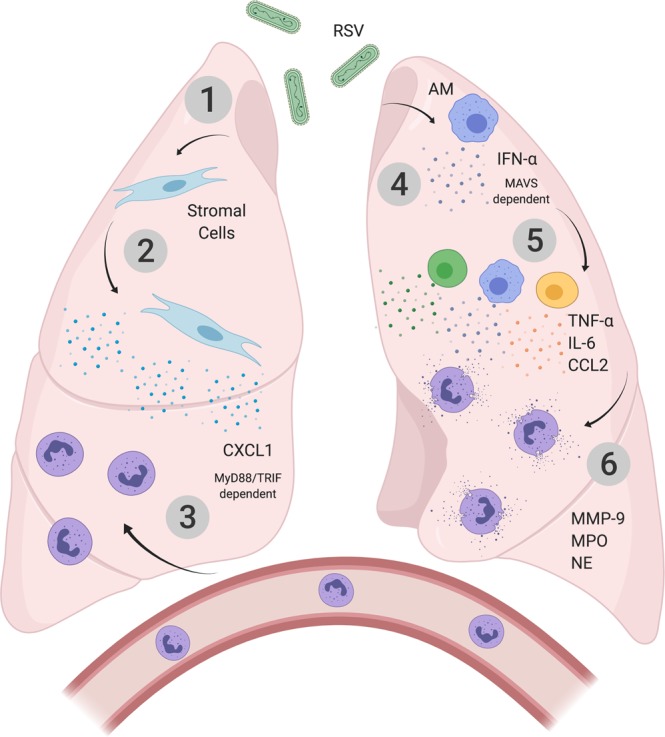
Schematic to illustrate the mechanism by which neutrophils are recruited and activated in the lung during RSV infection. RSV enters the airways (1) and interacts either directly or indirectly with stromal cells to induce the production of CXCL1 via MYD88/TRIF signaling (2). This drives the recruitment of neutrophils into the lungs from the vasculature (3). Meanwhile, AMs produce type I IFNs in response to RSV via signaling through MAVS (4). Type I IFNs drive the induction of other pro-inflammatory mediators, such as TNF-α and IL-6, and chemokines such as CCL2 by multiple other cell types including DCs and monocytes (5). When neutrophils encounter this pro-inflammatory environment, they upregulate cell surface CD64 and become activated to secrete MMP-9, MPO, and NE (6). The illustration was created using BioRender

Neutrophils are one of the first cell types to be recruited to the lung during RSV infection^21^ and lung neutrophilia is a hallmark of severe RSV infection in infants.^20,22,23^ Here, innate immune signaling deficient *Mavs*^*−/−*^ and *Myd88/Trif*^*−/−*^ mice were used to understand which RSV sensing pathways are required for the early recruitment and activation of lung neutrophils during RSV infection. Interestingly, we found that both MyD88/TRIF and MAVS signaling are required but that they control distinct aspects of the neutrophilic response (Fig. 7). Indeed, while MyD88/TRIF signaling was necessary for neutrophils to infiltrate the lung, it was dispensable for their activation whereas the opposite was true for the MAVS pathway. To our knowledge, this is the first study to demonstrate that distinct innate immune signaling pathways synergize to drive lung neutrophilia and neutrophil activation during a respiratory virus infection.

Neutrophils are classically considered rapid responders and, consistent with that, early neutrophil recruitment was detected in the lung post RSV infection. To assess the activation status of lung neutrophils, the cell surface expression of known activation markers was investigated by flow cytometry. This provides a sensitive readout of neutrophil activation as the expression level on each cell can be assessed individually. For a more global and functional readout of neutrophil activation in the lung the accumulation of MMP-9, MPO and NE in BAL fluid was assessed. NE is exclusively produced by neutrophils and our data in RSV-infected mice support clinical observations of increased NE in the nasal lavage of children with RSV-induced bronchiolitis.^23^ Neutrophil depletion experiments confirmed that neutrophils are the major source of secreted MMP-9 and NE in response to RSV. However, neutrophil depletion did not completely abolish airway MPO during the infection, suggesting there may be an alternative cellular source of this mediator. It is possible that monocytes also secrete MPO in the lung in response to RSV as human monocytes have been shown to contain moderate levels of intracellular MPO.^42^ Neutrophil granules contain >1200 unique proteins^43^ and it will be of interest to further investigate which additional factors are secreted during RSV infection. Of the cell surface activation markers tested, only CD64 (high affinity FcγRI) upregulation occurred on lung neutrophils specifically during RSV infection and not mock infection CD64 upregulation is associated with neutrophil activation in bacterial infections,^36^ however the functional relevance of CD64 cell surface expression on neutrophils in viral infections is not known. Interestingly, CD69 was upregulated on airway neutrophils during influenza infection^37^ but remained low during RSV infection, suggesting that neutrophils may respond differently to these two respiratory viruses.

Signaling via MyD88/TRIF was essential for neutrophil recruitment to the RSV infected lung (shown here and in *Myd88*^*−/−*^ mice^41^). MyD88/TRIF signaling has also been shown to be required for neutrophil recruitment during infection with influenza virus, *Staphylococcus aureus* and *Streptococcus pneumoniae*, reflecting TLR-dependent signaling or signals from IL-1 receptor family members, which also employ MyD88.^8,44,45^ However, it is not known whether the lung cell types involved, and the receptors used for initiating the neutrophil recruitment, are the same for different infections. For example, during *S. pneumoniae* infection, both hematopoietic and non-hematopoietic lung cells contribute to the production of neutrophil chemoattractants via MyD88 signaling.^44^ Furthermore, TLR signaling in non-hematopoietic cells drives neutrophil recruitment during influenza virus infection.^8^ During RSV infection, we found that non-epithelial, non-endothelial stromal cells (CD45^-^, EpCAM^-^, CD31^-^ cells) are the major producers of CXCL1 and that this production is dependent on signaling via MyD88/TRIF. The cell type in this mix of “other stromal cells” which is required for neutrophil recruitment, the receptors involved, the signaling which occurs downstream of MyD88/TRIF and the production of other neutrophil chemoattractants remain to be elucidated.

While neutrophil recruitment to the lung was not dependent on signaling via MAVS, concordant with previous data,^27^ activation was dependent on a functional MAVS signaling pathway. Neutrophils in the *Mavs*^*−/−*^ mice did not become activated during RSV infection, despite these mice having a higher viral load than wt mice. *Myd88/Trif*^*−/−*^ mice did not recruit neutrophils during RSV infection, yet this did not impair the ability of the host to control viral replication. We have previously demonstrated that impaired type I IFN production, via loss of MAVS signaling or type I IFN receptor signaling, results in increased viral loads,^21,27^ and that viral control is reestablished when mice are treated with CCL2 to restore the recruitment of anti-viral monocytes.^27^ Together, these studies suggest that type I IFNs, acting in part via monocytes, are the main drivers of early protection during RSV infection and that neutrophil recruitment and activation in the lung are dispensable for viral control.

While we cannot completely exclude that neutrophil activation requires MAVS signaling in neutrophils themselves, it is not thought that neutrophils can be activated directly by RSV particles.^46^ During RSV infection, MAVS signaling initiates type I IFN production and the amplification of type I IFN signaling via the IFNAR receptor drives a downstream signaling cascade that leads to the production of many anti-viral ISGs and pro-inflammatory mediators.^21,27^ Cytokines such as IFN-γ and GM-CSF, which were induced when RSV-infected *Mavs*^*−/−*^ mice were treated with rIFN-α, can activate neutrophils.^47^ When the pro-inflammatory environment was restored in RSV-infected *Mavs*^*−/−*^ mice by treatment with rIFN-α, neutrophils displayed an activated phenotype as measured by the upregulation of CD64 and by the secretion of MPO in the airways. However, these mice did not display significantly more MMP-9 and NE levels in BAL than mock-treated *Mavs*^*−/−*^ mice. It is possible that this may be a quantitative defect due to the fact that a single administration of IFN-α at 6 h p.i. may not sufficiently replicate the kinetics of IFN-α production in wt mice. Alternatively, we cannot exclude the possibility that factors in addition to IFN-α are required to drive full neutrophil activation. Therefore, our data suggest that *Mavs*^*−/−*^ deficient neutrophils become at least partially activated when they are in an inflammatory environment induced downstream of IFN-α. This likely involves ISG protein products upregulated downstream of type I IFN receptor signaling.^27–29^ It is interesting to consider that while type I IFNs have a beneficial inhibitory effect on viral load they also drive lung inflammation^21^ and, as we show in this work, neutrophil activation during RSV infection. Therefore, the potential use of type I IFNs as a therapeutic in RSV infection must be carefully considered to avoid immunopathology.

As RSV infected *Myd88/Trif*^*−/−*^ mice have intact MAVS signaling, we hypothesized that RSV infection would induce the upregulation of the factor/s which are required for neutrophil activation. Indeed, the very few lung neutrophils found in *Myd88/Trif*^*−/−*^ mice upregulated CD64. We suspect that the reason MMP-9, MPO and NE were not detected in the airways of these mice was because there were too few neutrophils to release detectable quantities of these mediators. Consistent with that possibility, when neutrophils were artificially recruited into the lungs of RSV-infected *Myd88/Trif*^*−/−*^ mice by administration of rCXCL1, the lung neutrophils upregulated CD64 and MMP-9, MPO and NE were detected in the airways. It is known that CXCL1 has a role in both the recruitment and activation of neutrophils^35^ and treatment with rCXCL1 on its own did induce some production of MMP-9, MPO and NE in the lungs of *Myd88/Trif*^*−/−*^ mice. However, rCXCL1 treatment in the absence of RSV did not induce the upregulation of CD64 on lung neutrophils. Furthermore, neutrophil mediator production was amplified and CD64 upregulated on neutrophils by RSV infection during rCXCL1 treatment of *Myd88/Trif*^*−/−*^ mice, demonstrating that the inflammatory environment induced by RSV infection was required for full neutrophil activation. These data support our finding that an intact MAVS signaling pathway is sufficient to induce an inflammatory environment that at least partially activates neutrophils during RSV infection.

To summarize, this paper demonstrates for the first time that two distinct innate immune signaling pathways collaborate to ensure that both neutrophil recruitment and activation occur during RSV infection. MyD88/TRIF signaling is necessary to attract and recruit neutrophils and MAVS signaling is crucial to create an inflammatory environment in the lung that drives at least partial neutrophil activation. Excessive neutrophilia can be detrimental to the health of lung tissue and compromise efficient gas exchange. As such, a deeper understanding of the molecular pathways that regulate neutrophil recruitment and activation may help pave the way towards development of potential therapies.

## Methods

### Mice

Wt C57BL/6 mice were purchased from Charles River. *Ifna6gfp*^+/−^, *Ifna6gfp*^+/−^ *Myd88Trif*^*−/−*^ and *Ifna6gfp*^+/−^ *Mavs*^*−/−*^ mice were bred in-house (obtained from S. Akira, Japan). The GFP signal has not been quantified in this work so the mice will be denoted as wt, *MyD88/Trif*^*−/−*^ and *Mavs*^*−/−*^ mice, respectively. All mice were bred and housed in specific pathogen-free conditions and were gender and age-matched (7–12 weeks). Different strains of mice were not co-housed but kept in the same breeding room. The breeders of *MyD88/Trif*^*−/−*^ mice were kept on Septrin in the drinking water because of their immunocompromised status. All animal experiments were reviewed and approved by the Animal Welfare and Ethical Review Board (AWERB) within Imperial College London and approved by the UK Home Office in accordance with the Animals (Scientific Procedures) Act 1986 and the ARRIVE guidelines.

### Virus and infections

Plaque-purified human RSV (originally A2 strain from ATCC, US) was grown in Hep2 cells.^48^ RSV was inactivated by exposing virus to UV light for 2 min (UV RSV) in a CX-2000 UV cross-linker (UVP). Mice were lightly anaesthetized before administration intranasally (i.n.) of 100 µl containing 10^6^ FFU RSV or PBS control. In some instances, this was followed by instillation of recombinant proteins (1 µg IFN-α (Miltenyi Biotec), 10 µg CXCL1 (Biolegend)) or a protein control, bovine serum albumin (BSA; Sigma Aldrich). Mice were sacrificed post-infection (p.i.) by a fatal dose of pentobarbital injected intraperitonially (i.p.).

### Antibody mediated neutrophil depletion

Mice were given 200 µg i.n. and 500 µg i.p. anti-Ly6G MAb or isotype rat Ig2A MAb (Bio X Cell) 1 day pre-infection.^49,50^

### Airway cell processing

To recover the airway cells and immune mediators, BAL was performed. One ml PBS supplemented with 0.5 mM EDTA was used to flush the lungs three times. The BAL fluid was centrifuged and supernatant stored at −80 °C. Red blood cells (RBCs) were removed by lysis with ACK (0.15 M NH_4_Cl, 1.0 mM KHCO_3_, 0.1 mM Na_2_EDTA). The cell number was determined by Trypan Blue (Thermo Fisher Scientific) exclusion of dead cells. BAL cells were termed airway cells throughout. Airway cells were either stained for flow cytometry or the cellular composition was determined by spinning 1–2 × 10^5^ cells onto a microscope slide (Thermo Scientific) at 450 rpm for 5 min using Cytospin 4 Cytocentrifuge (Thermo Fisher Scientific). Slides were H&E stained using Reastain Quick-Diff kit (Gentaur), according to the manufacturer’s instructions. Cells were classified as neutrophils using a microscope and ≥300 total cells were counted.

### Isolation of lung cells and peripheral mononuclear blood cells

For RNA extractions, lung lobes were snap-frozen in liquid nitrogen and stored at −80 °C. For flow cytometry, 1–3 lung lobes were collected in complete DMEM (cDMEM; supplemented with 10% fetal bovine serum, 2 mM L-glutamine, 100 U/ml penicillin, and 100 μg/ml streptomycin). Collagenase D (1 mg/ml; Roche) and DNase I (30 µg/ml; Invitrogen) was added and samples processed with a gentle MACS dissociator (Miltenyi Biotech). Lungs were incubated shaking at 37 °C for 1 h and then processed again with a gentle MACS dissociator. RBCs were removed by ACK lysis. Cells were re-suspended in PBS for flow cytometry and filtered through a 100 µM cell strainer (Greiner BioOne). The lung cell count was quantified by Trypan Blue (Thermo Fisher Scientific) exclusion of dead cells. At least 75 µl blood was collected in 1 ml PBS supplemented with 5 mM EDTA. RBCs were removed by lysis with ACK. Cells were re-suspended in FACS buffer (PBS supplemented with 1% BSA, 0.05 mM EDTA) before flow cytometry staining.

### Flow cytometry

For flow cytometry staining, 2.5 × 10^6^ lung cells were incubated for 20 min at 4 °C with a purified rat IgG2b anti–mouse CD16/CD32 receptor antibody (BD) to block Fc binding in FACS buffer. For surface staining, cells were stained with fluorochrome-conjugated antibodies against CD45 (30-F11, BV605), CD11b (M1/70, AF700), CD64 (X54-5/7.1, APC), Ly6G (1A8, BV570/BV785), CD3ε (145-2C11, FITC), Ly6C (HK1.4, BV421), CD19 (6D5, FITC), CD69 (H1.2F3, Per CP Cy5.5), MHC-II (M5/114.15.2, APC eF870), CD62L (MEL-14, BV421), CD182 (SA044G4, PE), CD11c (HL3, PE-CF594) for 25 min at 4 °C. Cells were washed with PBS and stained with fixable live-dead Aqua dye (Invitrogen) for 30 min. Cells were fixed by incubation with 100 µl 1% paraformaldehyde or Cytofix™ Fixation Buffer (BD) for 20 mins at 4 °C and stored in FACS buffer. Analysis was performed on a BD LSR Fortessa. Acquisition was set to 250,000 single, live, CD45^+^ cells. All antibodies were purchased from BD, BioLegend, or eBioscience. Data were analyzed with FlowJo software (Tree Star). Total cell populations were quantified as the whole lung count × (%population of CD45^+^ cells) × (%CD45^+^ cell of live cells) × (proportion of lung tissue sampled).

### FACS

For cell sorting of lung cell populations, single cell suspensions were obtained by dispase digestion (5 mg/ml; Roche) and DNase I (250 μg/ml; Sigma), as reported.^27^ Cells were incubated with a purified rat IgG2b anti–mouse CD16/CD32 receptor antibody (BD) and stained with fluorochrome-conjugated antibodies against CD11c (HL3, PE-CF594), CD31 (390, PE), CD45 (30-F11, APC-Cy7), and EpCAM (G8.8, PerCP/Cy5.5) as described above. Sytox Blue Dead Cell Stain (1:8000; Life Technologies, UK) was added to cells before running on a BD FACSAria III using FACSDiVa software (BD Bioscience). Sorted cells were stored in Trizol™ Reagent (Invitrogen, UK) until RNA extraction was performed.

### Fluorescent microscopy

Lungs were inflated with 1 ml 50% OCT (VWR) diluted in PBS and frozen on dry ice. Ten micrometers lung cryosections were cut using a cryostat (Bright OTF5000 LS) and sections were fixed in acetone for 10 min at room temperature. Sections were rehydrated twice in PBS before blocking with purified rat IgG2b anti–mouse CD16/CD32 receptor antibody (BD) to block Fc binding in FACS buffer for 30 min at room temperature. To detect Ly6G^+^ neutrophils, sections were stained with α-Ly6G 1A8 (1:800, Abcam) overnight at 4 °C. Sections were then washed twice in PBS and stained with a species-specific secondary antibody conjugated to Alexa Fluor 647 (1:200, Abcam) for 2 h in the dark at 4 °C. Sections were washed twice in PBS and coverslips were mounted into glass slides with ProLong® Gold Anti-fade Mountant with DAPI (ThermoFisher Scientific). The Zeiss Inverted Widefield Microscope on a 20× dry lens was used for obtaining images of the cells. Analysis of the images was performed on Fiji, an open-source imaging processing software (ImageJ).

### RNA extraction and quantitative RT-PCR

Lungs were homogenized using a TissueLyser LT (Qiagen) and total RNA was extracted from the lung tissue supernatant using RNeasy Mini kit (Qiagen). RNA extraction from sorted lung cells was performed using Trizol (Invitrogen). Following the chloroform step, the aqueous phase containing RNA was further processed using the RNeasy Mini or Micro Kit according to manufacturer’s instructions (Qiagen). RNA concentration was determined by NanoDrop (Thermo Scientific). In all, 2 µg or 9 µl (sorted cells) of RNA was converted to cDNA using High Capacity RNA-to-cDNA kit (Applied Biosystems). RT-qPCR was performed with Quantitect Probe PCR Master Mix (Qiagen). For mRNA analysis of *Gapdh, Cxcl1, Cxcl2, Il6*, *Csf2*, and *Ifna5* gene specific primers and probes were used (all Applied Biosystems). For absolute quantification of RSV L gene, TNF-α and IFN-γ mRNA, the exact number of copies of the gene of interest was calculated using a plasmid DNA standard curve for each gene and normalized to *Gapdh*.^21^ RT-qPCR was performed using the 7500 Fast Real-Time PCR System (Applied Biosystems). To quantify relative mRNA expression the mean ΔCT was calculated for each target gene relative to *Gapdh* (encoding glyceraldehyde-3-phosphate dehydrogenase) and expressed as 2^−ΔCT^. Analysis was performed using 7500 Fast System SDS Software (Applied Biosystems).

### Immune mediator detection

ELISA was used to measure the concentration of CXCL1, MMP-9, MPO, NE (all DuoSet ELISA kits from R&D systems), IL-6^21^ and IFN-α.^27^ Absorbance was determined at 450 nm, on SpectraMax Plus (Molecular Devices) or FLUOstar Omega (BMG Labtech) plate readers and analyzed using SoftMax (Molecular Devices) or Mars (BMG Labtech) software.

### Statistical analysis

Statistical analysis was performed using Prism (GraphPad software) version 6. Data are presented as the mean ± SEM. As indicated, one-way ANOVA followed by Tukey’s post hoc was used to compare multiple groups. To compare genotypes during mock and RSV infection, a two-way ANOVA, followed by either Bonferroni’s or Tukey’s post hoc test, was used as indicated. *P* values < 0.05 were considered statistically significant for all tests. *P < 0.05; **P < 0.01; ***P < 0.001.

## Supplementary information

Supplementary Information
