# Supplementary material for: Neutrophil recruitment and activation are differentially dependent on MyD88/TRIF and MAVS signaling during RSV infection
Source: Mucosal Immunol. 2019 Jul 29;12(5):1244–55. doi: 10.1038/s41385-019-0190-0 (PMC6778055; doi:10.1038/s41385-019-0190-0)
Supplement: Supplementary file 1 — Supplementary Information [file 41385_2019_190_MOESM1_ESM.pdf]

Neutrophil recruitment and activation are independently regulated by MyD88/TRIF and MAVS signaling during RSV infection.
Kirsebom et al.

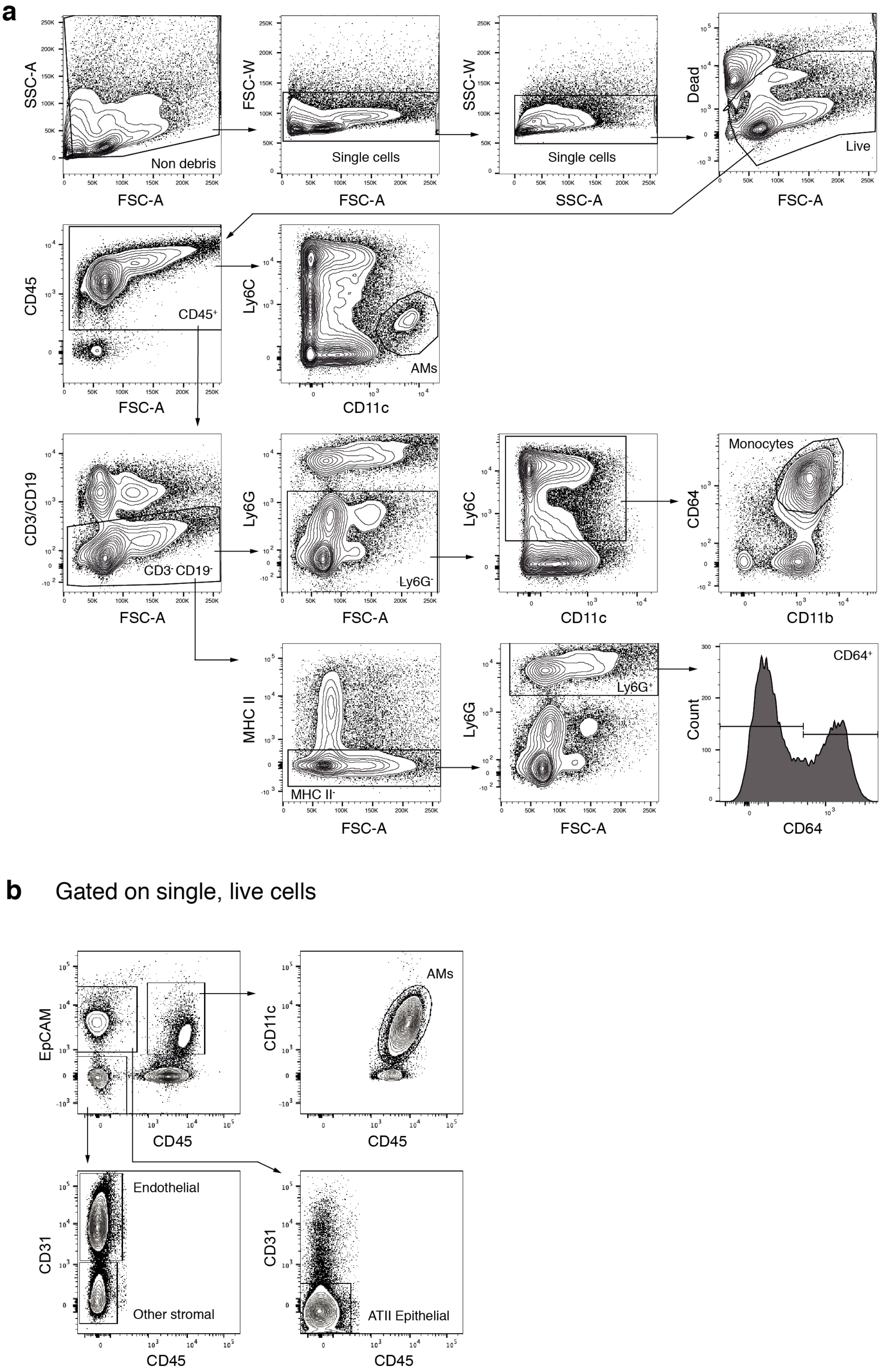

Supplementary Figure 1. Gating strategies for identifying airway and lung innate immune cells and stromal cells. Mice were intranasally infected with RSV. **a.** Airway/lung innate immune cells were obtained by collagenase digestion and stained for the indicated cell surface molecules. After excluding debris and gating on single, live, $CD45^+$ cells, the depicted gates were used to identify AMs, monocytes and neutrophils. $Ly6G^+$ neutrophils were further gated to determine cell surface activation marker expression. **b.** Lung cells, including stromal cells, were obtained by dispase digestion and stained for the indicated cell surface molecules. After excluding debris and gating on single and live cells, the depicted gates were used to identify and sort AMs, ATII epithelial cells, endothelial cells and other stromal cells.

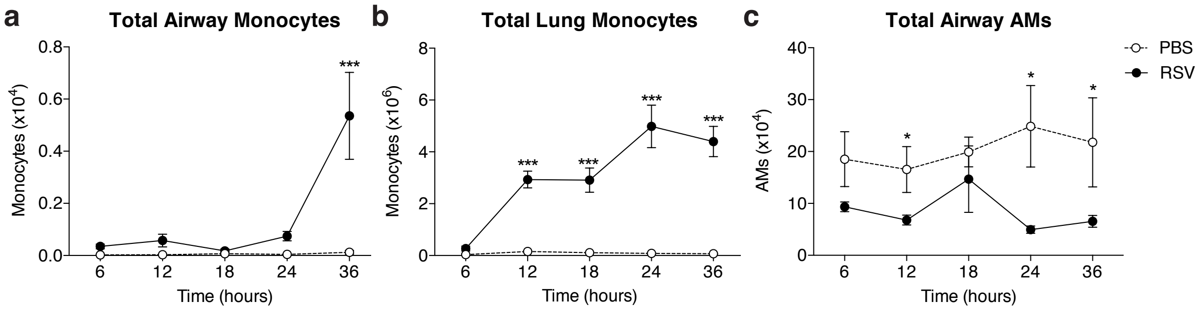

Supplementary Figure 2. AMs and monocyte recruitment in the lung during RSV infection*.* Wt mice were intranasally mock (PBS) or RSV infected. **a-b.** Total numbers of $CD45^+$, $CD3^-$, $CD19^-$, $Ly6G^-$, $Ly6C^+$, $CD64^+$, $CD11b^+$ monocytes were quantified in the airways and lung using flow cytometry at the indicated time-points. **c.** Total numbers of $CD45^+$, $Ly6C^+$, $CD11c^+$ AMs were quantified in the airways using flow cytometry at the indicated time-points. Data are presented as the mean±SEM of 3-5 (PBS), or 8-13 (RSV) individual mice, pooled from at least two independent experiments. Statistical significance of differences between mock and RSV infection was determined by one-way ANOVA with Tukey's post hoc test. * P ≤ 0.05, *** P ≤ 0.001.

Neutrophil recruitment and activation are independently regulated by MyD88/TRIF and MAVS signaling during RSV infection.
Kirsebom et al.

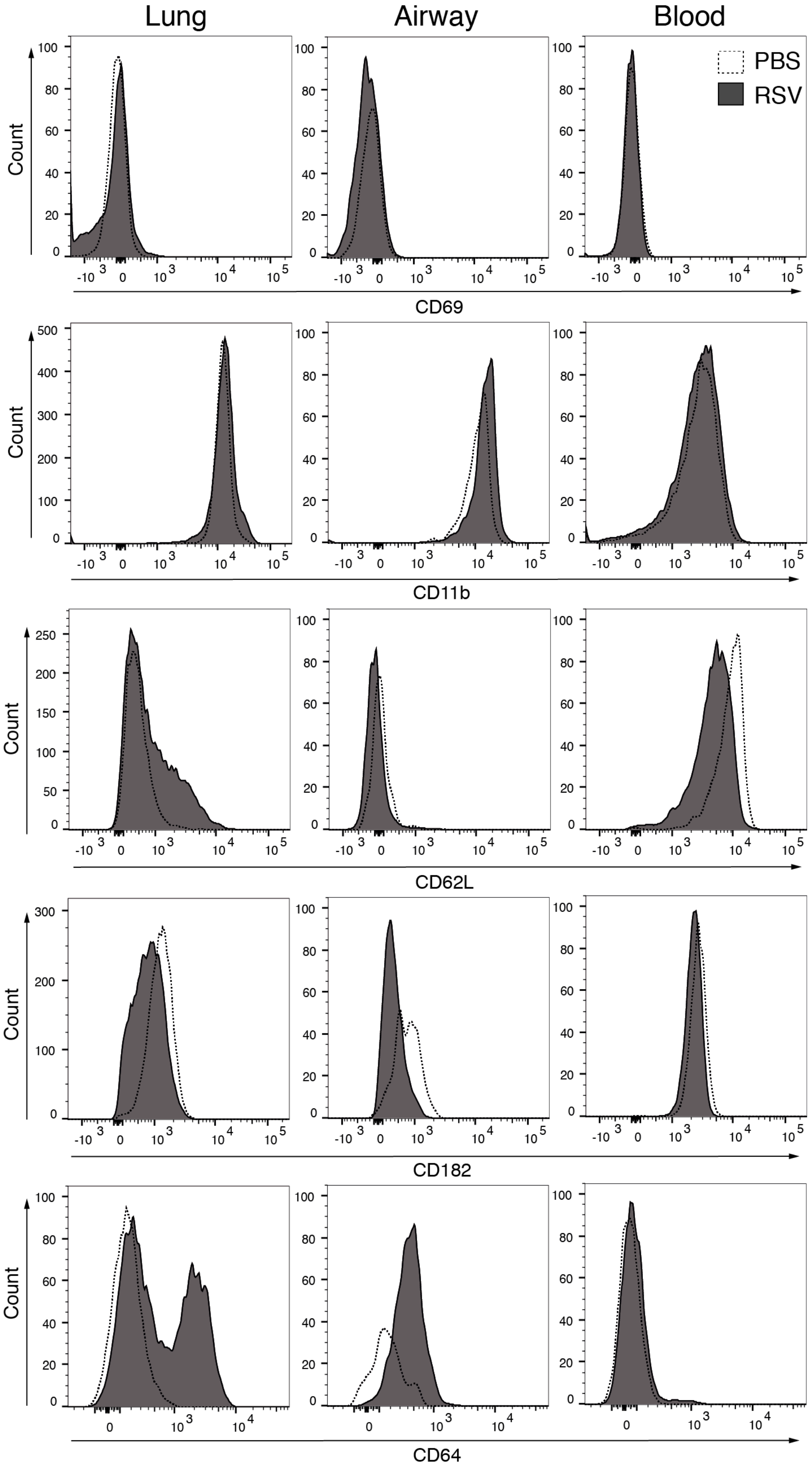

Supplementary Figure 3. CD64 upregulation is a marker of lung neutrophil activation during RSV infection. C57BL/6 mice were mock (PBS) or RSV infected for 18h. Representative histograms of CD69, CD11b, CD62L, CD182 and CD64 expression on $CD45^{+}$, $CD3^{-}$, $CD19^{-}$, $MHC\text{-}II^{-}$, $Ly6G^{+}$ neutrophils in the lung, airways and blood.

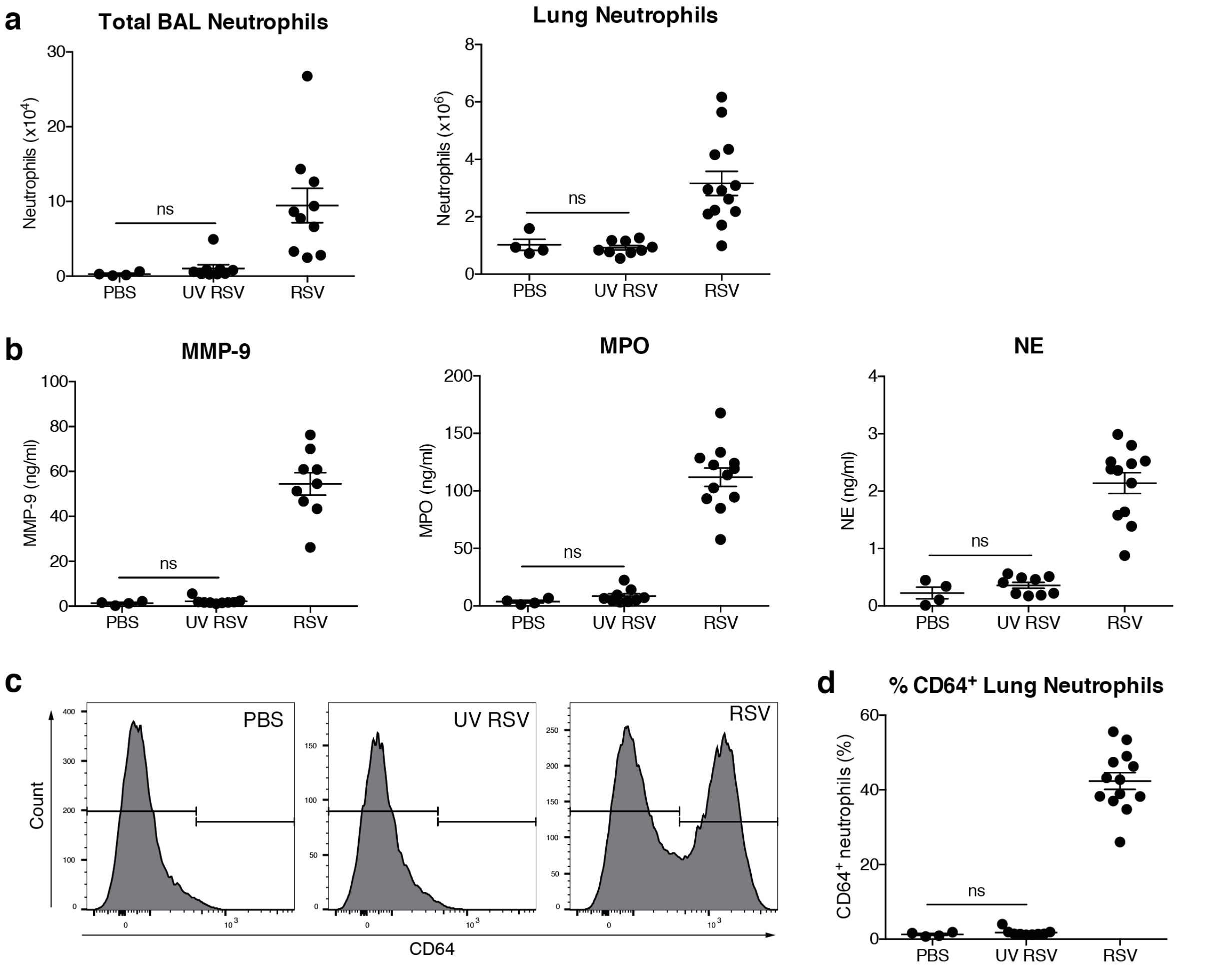

Supplementary Figure 4. Live virus is necessary for neutrophil recruitment and activation during RSV infection. C57BL/6 mice were mock (PBS), UV RSV or RSV infected for 18h. **a.** Total numbers of $CD45^+$, $CD3^-$, $CD19^-$, MHC-II^-, $Ly6G^+$ neutrophils were quantified in the airways and lung using flow cytometry. **b.** MMP-9, MPO and NE were detected in the BAL fluid by ELISA. **c.** Representative histograms of CD64 expression on $CD45^+$, $CD3^-$, $CD19^-$, $Ly6G^+$ neutrophils in the lung. **d.** Frequency of $CD64^+$ lung neutrophils, as quantified by flow cytometry. Data are presented as the mean±SEM of 4 (PBS), 9 (UV RSV) and 10-13 (RSV) individual mice, pooled from two independent experiments. Statistical significance of differences was determined by one-way ANOVA with Tukey's post hoc test. Only the statistical significance between PBS and UV RSV is shown.

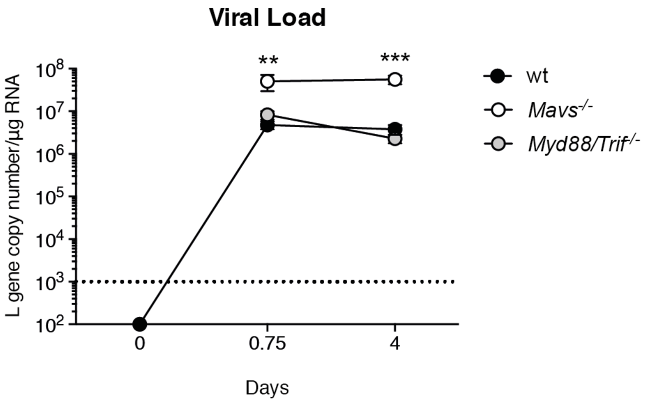

Supplementary Figure 5. MAVS signaling, but not MyD88/TRIF, is required to inhibit viral replication during RSV infection *in vivo*. Wt, *Mavs*$^{-/-}$ and *Myd88/Trif*$^{-/-}$ mice were intranasally mock (PBS) or RSV infected for 18h or 4 days. RSV L gene copy number was quantified from lung tissue by RT-qPCR. The dotted line represents the detection limit. Data are presented as the mean±SEM of 10 (PBS) and 8-10 (RSV) individual mice, pooled from two independent experiments. Statistical significance of differences between RSV infected mice was determined by two-way ANOVA with Tukey's post hoc test. ** P ≤ 0.01, *** P ≤ 0.001.

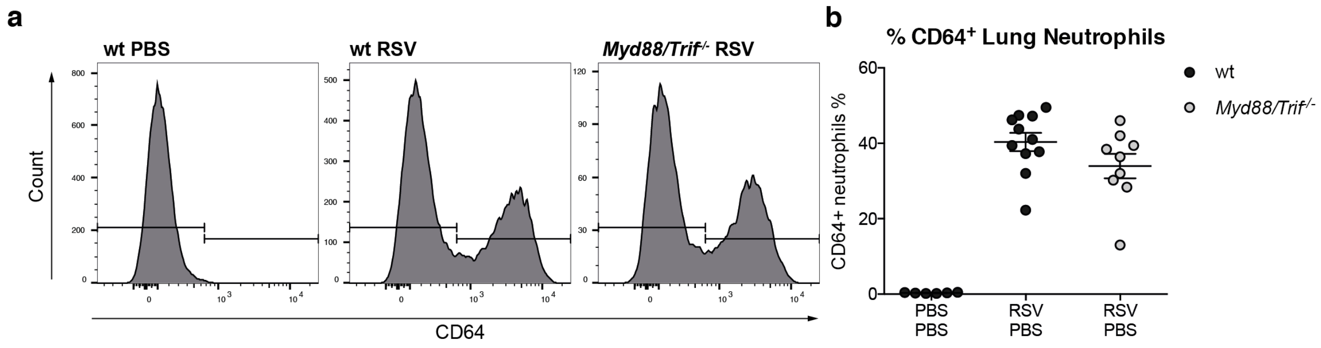

Supplementary Figure 6. Wt and *Myd88/Trif*$^{-/-}$ mice were mock (PBS) or RSV infected for 18h. **a.** Representative histograms of CD64 expression on CD45^{+}, CD3^{-}, CD19^{-}, MHC II^{-}, Ly6G^{+} neutrophils in the lung. **b.** Frequency of CD64^{+} lung neutrophils, as quantified by flow cytometry. Data are presented as mean±SEM of 6-12 mice from each group (represented as individual dots), pooled from 2-3 independent experiments. Statistical significance of differences was determined by one-way ANOVA with Tukey's post hoc test.

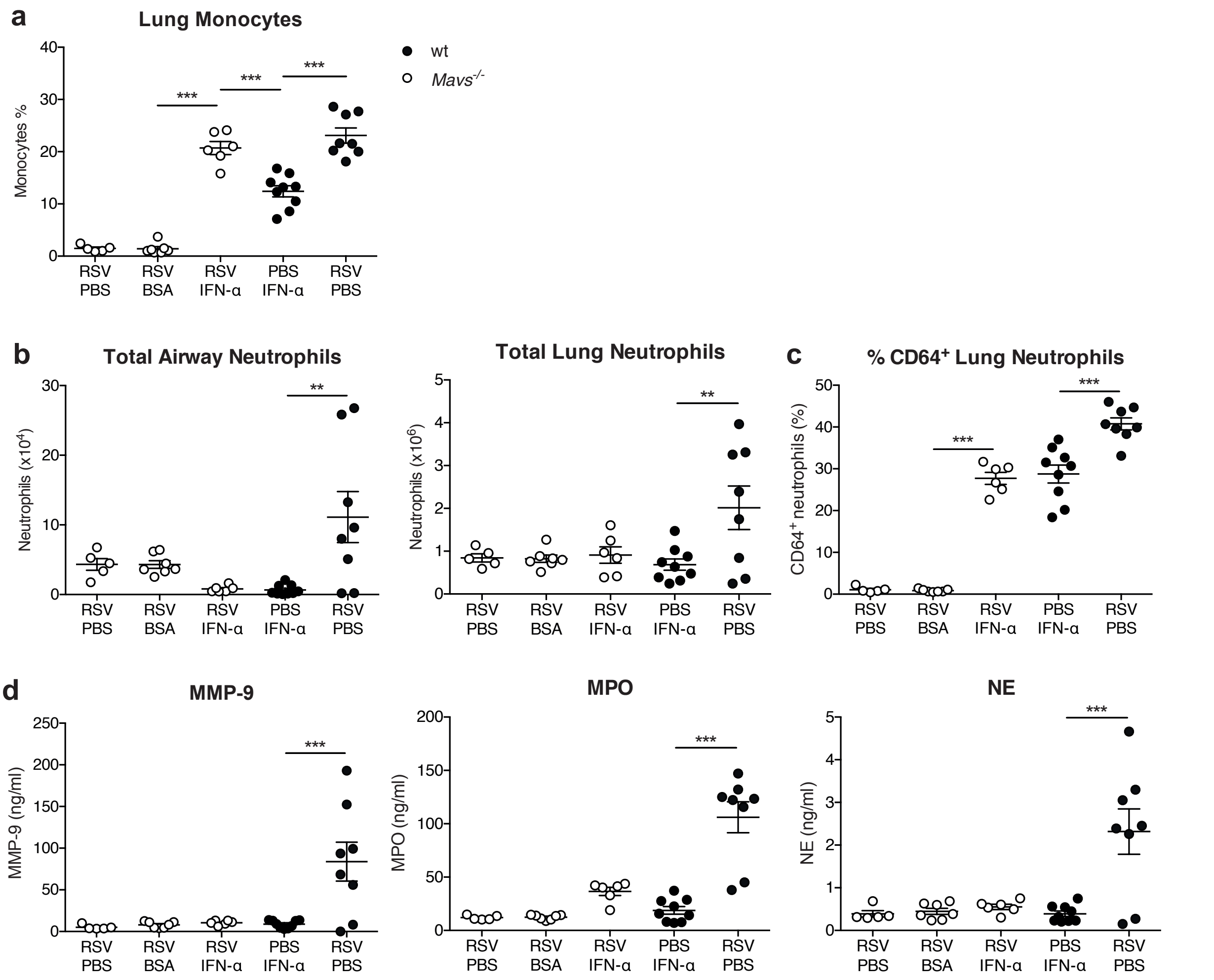

Supplementary Figure 7. 1 µg rIFN-α is sufficient to create a pro-inflammatory environment in the lung. Wt and *Mavs*$^{-/-}$ mice were mock (PBS) or RSV infected for 24h. PBS, 1 µg rIFN-α or 1 µg BSA was administered i.n. 6h p.i. **a.** CD45^{+}, CD3^{-}, CD19^{-}, Ly6G^{-}, Ly6C^{+}, CD64^{+}, CD11b^{+} monocytes were quantified in the lung using flow cytometry. **b.** Total CD45^{+}, CD3^{-}, CD19^{-}, MHC II^{-}, Ly6G^{+} neutrophils were quantified in the airways and in the lung. **c.** Frequency of CD64^{+} lung neutrophils, as quantified by flow cytometry. **d.** MMP-9, MPO and NE were detected in the BAL fluid by ELISA. Data are presented as the mean±SEM of 5-9 individual mice, pooled two independent experiments. Statistical significance of differences was determined by one-way ANOVA with Tukey's post hoc test. ** P ≤ 0.01, *** P ≤ 0.001.
